# Principles and correction of 5’-splice site selection

**DOI:** 10.1080/15476286.2022.2100971

**Published:** 2022-07-22

**Authors:** Florian Malard, Cameron D Mackereth, Sébastien Campagne

**Affiliations:** Inserm U1212, CNRS UMR5320, ARNA Laboratory, University of Bordeaux, Bordeaux Cedex, France

**Keywords:** RNA splicing, 5’-splice site, U1 snRNP, antisense oligonucleotides, splicing modifiers

## Abstract

In Eukarya, immature mRNA transcripts (pre-mRNA) often contain coding sequences, or exons, interleaved by non-coding sequences, or introns. Introns are removed upon *splicing*, and further regulation of the retained exons leads to alternatively spliced mRNA. The *splicing* reaction requires the stepwise assembly of the spliceosome, a macromolecular machine composed of small nuclear ribonucleoproteins (snRNPs). This review focuses on the early stage of spliceosome assembly, when U1 snRNP defines each intron 5’-splice site (5ʹss) in the pre-mRNA. We first introduce the splicing reaction and the impact of alternative splicing on gene expression regulation. Thereafter, we extensively discuss splicing descriptors that influence the 5ʹss selection by U1 snRNP, such as sequence determinants, and interactions mediated by U1-specific proteins or U1 small nuclear RNA (U1 snRNA). We also include examples of diseases that affect the 5ʹss selection by U1 snRNP, and discuss recent therapeutic advances that manipulate U1 snRNP 5ʹss selectivity with antisense oligonucleotides and small-molecule splicing switches.

## Introduction

In the three kingdoms of life, information is encoded in DNA molecules that form the genomes of membrane-based organisms [1]. Archaea, Bacteria and Eukarya share the use of RNA molecules as obligatory mediators of DNA gene expression [1]. RNA molecules are transcribed from genomic DNA and are usually further processed into various categories of mature RNA molecules. RNA classification distinguishes between messenger RNAs (mRNAs) that code for proteins, and noncoding RNAs (ncRNAs) that do not encode proteins [2,3]. Each mRNA molecule is transcribed from a genomic DNA gene, and this is true for Prokarya (i.e. Archaea, Bacteria) and Eukarya. In the early days of RNA biology, a major finding was that mRNA precursors from eukaryotic organisms and viruses may contain intervening sequences that are removed from the immature mRNA transcript (pre-mRNA) to yield a mature mRNA [4–6]. Terminological rules emerged and intervening sequences were termed as *introns*, while their removal from the pre-mRNA produced a functional mRNA consisting of joined *exons*. This maturation process occurs within the nucleus and is defined as RNA *splicing* [7]. This discovery was therefore the basis for investigation of the discontinuous nature of eukaryotic DNA genes, which contrasted with the continuous organization of RNA-coding information in prokaryotic organisms [8]. It is now clear that eukaryotic protein coding genes are generally organized as a succession of exons that code for the sequence of amino acids, and are spaced by introns that do not code for amino acids. Nevertheless, long-standing debates remain on the origin of the eukaryotic gene structure [9,10]. Overall, the splicing reaction is a crucial maturation step by which non-coding sequences are spliced out from the pre-mRNA to yield a functional mRNA [11].

In this review, we briefly introduce the splicing reaction and machinery, and describe how it is important for gene expression and regulation. On this basis, we focus on the molecular details by which the U1 small nuclear ribonucleoprotein (snRNP) defines the intron 5’-splice site (5ʹss) at one end of the intron during pre-mRNA processing. We describe examples in which this 5ʹss selection is perturbed in a variety of diseases, leading to aberrant splicing and the production of a suboptimal to non-functional mRNA. Finally, we discuss recent therapeutic advances to manipulate U1 snRNP 5ʹss selectivity by using synthetic splicing switches.

### Splicing description

**Figure 1.**
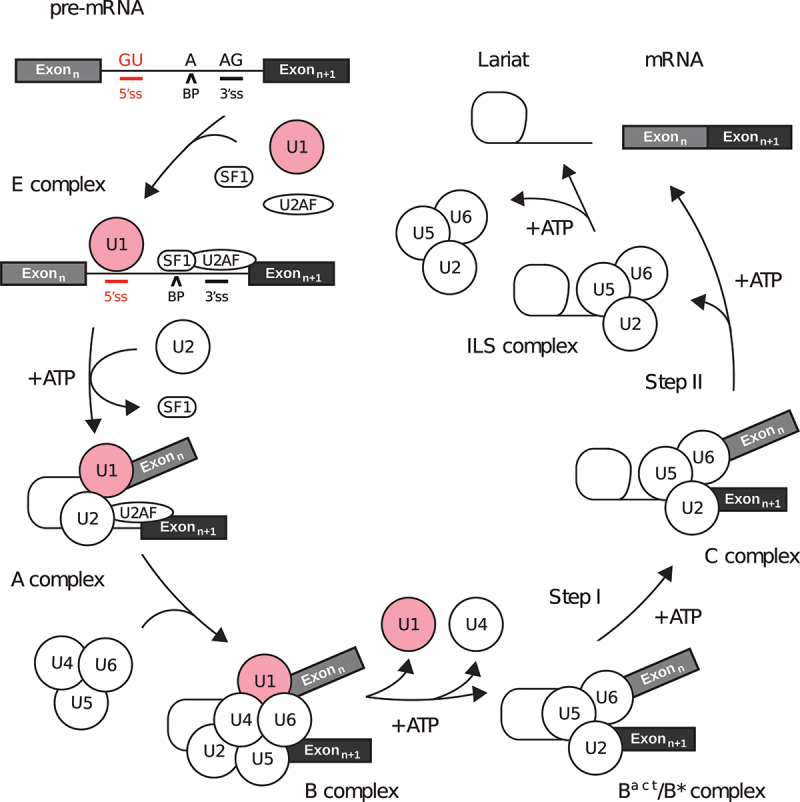
Overview of the splicing reaction. The pre-mRNA must contain at least two exons (boxes) interleaved by one intron (black line) to be relevant for splicing. A single intron is defined by a pair of 5’- and 3’-splice sites (5ʹss, 3ʹss), as well as the branch point (BP) adenosine. Five small nuclear ribonucleoproteins (snRNPs) are the core components of the spliceosome machinery (U1/2/4/5/6) which requires auxiliary factors such as SF1 and U2AF. For each step in the diagram, the name of the corresponding spliceosomal complex is given.

The overall splicing reaction relies on a highly dynamic macromolecular machine called the spliceosome (Fig. 1) [11]. More than 150–300 proteins enter and exit the spliceosome with each step of the splicing cycle [12,13]. The major spliceosome requires five functional subunits known as small nuclear ribonucleoproteins (snRNPs): each is defined by a snRNP-specific small nuclear RNA (snRNA) to which common and snRNP-specific proteins are bound [14]. Given a prototypical pre-mRNA segment, represented here as exon_n_–intron–exon_n+1_, the splicing reaction is defined by the presence of a 5’-splice site (5ʹss) – or donor splice site – at the exon_n_–intron junction, and by a 3’-splice site (3ʹss) – or acceptor splice site – at the intron–exon_n+1_ junction. In addition, the branch point (BP) is an intron motif upstream of the 3ʹss that includes an essential adenosine required for the splicing reaction (Fig. 1).

Spliceosome assembly is a stepwise process that begins with the binding of U1 snRNP, SF1 and U2AF proteins to the 5ʹss, branch point and 3ʹss, respectively. The binding of these essential splicing factors results in the formation of the E complex [15]. Next, U2 snRNP is recruited to the branch point by exchanging with SF1, where it interacts with U1 snRNP in an ATP-dependent manner to form the A complex. This leads to the pairing between the splice sites [16]. The tri-snRNP (U4/U6.U5) composed of U4, U5 and U6 snRNP is next recruited by the A complex, displacing U1 snRNP downstream of the 5ʹss, and leading to formation of the pre-B complex [17]. RNP remodelling of the pre-B complex results in the dissociation of U1 snRNP and the pre-catalytic B complex [18]. Further remodelling events induce the release of U4 snRNP, leading to the activated B complex (B^act^) [19]. The resulting activated complex is converted into a catalytic pre-branching spliceosome (B*) that performs the first step of splicing. The essential adenine base within the branch point acts as a nucleophile, and attacks a guanine within the 5ʹss to form the spliceosomal C complex [20]. This reaction is followed by numerous RNP rearrangements that include a large-scale movement of U2 snRNP to result in the step II catalytically activated C* complex [21]. Splicing is completed upon ligation between the 3’-end of the cleaved 5ʹss and the 3ʹss, ultimately leading to exon–exon junction and release during the post-catalytic P complex, while the intron lariat to which U2, U5 and U6 remain bound is later released from the ILS complex [22]. In addition, research in multiple model organisms using various approaches have found that splicing is predominantly co-transcriptional, meaning that the spliceosome assembles on the nascent pre-mRNA during transcription [23,24]. Thanks to the cryo-EM revolution, most of the different spliceosome intermediates have now been studied at the atomic level, thus revealing the molecular mechanisms of the splicing reaction [25].

The nuclear spliceosome was also proposed to play a role in the splicing of mitochondrial RNA (mtRNA). The mitochondrial genome contains 13 protein-encoding genes that may contain group I/II introns. They harbour 5’ and 3’ boundaries similar to nuclei-like canonical splice sites and were proposed to be spliced out by the nuclear spliceosome [26]. Furthermore, the loss of U1 snRNP subunits shifts energy metabolism from glycolysis to OXPHOS in a cell-specific manner, in line with the implication of the nuclear spliceosome in the processing of mtRNAs [27].

### Alternative splicing

An intron is any segment within a gene that is removed upon splicing, and the 5’- and 3’-splice sites (5ʹss, 3ʹss) define the intron/exon boundaries. Conversely, an exon is any part of a gene that is a part of the mature mRNA. Due to the regulation of splice site recognition, the splicing reaction may create dynamic patterns through alternative splicing, and the underlying molecular regulatory mechanisms are still under investigation. At the start of the Human Genome Project in the 1990s, ~100,000 protein-coding genes were expected to be found [28,29]. In the following decade, estimations based on the consensus human genome revised this number to ~26,000 protein-coding genes, which has now shrunk to ~20000 upon refined analysis of the complete genome [30,31]. From an anterior perspective, this low number of human genes was unexpected when compared with the ~6000 genes that had been predicted from the genome of *Saccharomyces cerevisiae* (yeast) [32]. The question at that time was how this relatively small difference in the number of protein-coding genes may account for the incredible gap of complexity between yeast and human. This discrepancy was partially answered based on the observation that only ~3% of yeast genes contain introns, whereas more than 97% of human genes have introns [33]. In terms of intron frequency per gene, the average is less than 0.1 in yeast, while it is ~8 in humans, which is the highest value across eukaryotic species [34]. These important differences are correlated with the effective size of the reference proteome for each species: 6050 proteins for yeast (UniProtKB UP000002311) and 79038 proteins for human (UniProtKB UP000005640) [35]. In terms of gene:protein ratio, the average is 1:1 in yeast and 1:4 in humans, although this number does not account for the protein isoforms that remain to be experimentally detected.

The link between intron frequency and the number of proteins associated with one gene is explained at the transcript level by this idea of alternative splicing [36–39]. That is, a single pre-mRNA may produce several mature mRNA transcripts due to a variable exon composition and with striking consequences for the proteome diversity [38,39]. Consistently, the proportion of genes subject to alternative splicing is correlated with the level of complexity of the organism, reaching higher levels in primates [40]. From the perspective of the interactome, interaction profiling experiments have shown that the majority of isoform pairs share less than 50% of their interactions. Alternative splicing thus produces isoforms that behave more like distinct proteins rather than minor variants, which again expands the functional potential of a single gene [41]. Because alternative splicing is achieved by the selection of subsets of 5ʹss and/or 3ʹss in a competitive manner, a small number of mutations within these motifs can dramatically change exon-selection patterns. This property also suggests that alternative splicing is an important contributor to the appearance of novel phenotypes [42,43], and several studies suggest that alternative splicing contributes to accelerated evolutionary changes because it can create evolutionary *hotspots* within a protein while retaining the original protein sequence [44,45]. In this context, the U1 snRNP has a crucial role because it initiates the splicing reaction by defining the 5ʹss, hence the selection of alternative 5ʹss is directly linked with changes in U1 snRNP recruitment.

### Regulation of gene expression

**Figure 2.**
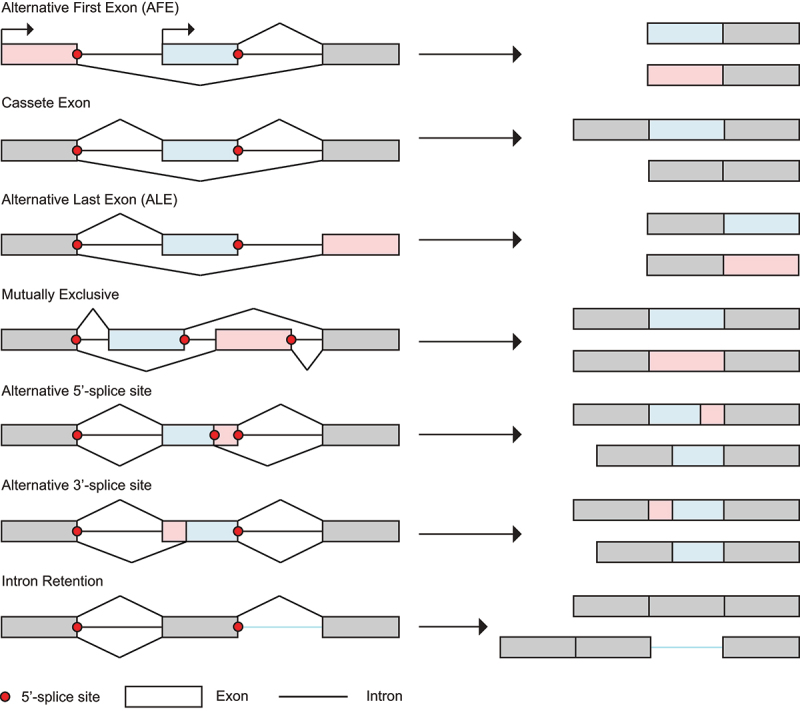
Alternative splicing events. Alternative splicing uses the intron 5’- and 3’-splice sites (3ʹss, 5ʹss), but with variations arising from different mechanisms: alternative first exon (AFE), alternative last exon (ALE), cassette exon, mutually exclusive exons, alternative 5ʹss and/or 3ʹss selection and intron retention.

Alternative splicing is an essential regulatory process that uses the intron 5’- and 3’-splice sites (5ʹss, 3ʹss), but with variation arising from distinct mechanisms: alternative first exon (AFE), alternative last exon (ALE), cassette exon, mutually exclusive exons, intron retention and alternative 5ʹss and/or 3ʹss selection (Fig. 2) [46–50]. Alternative splicing relies on alternative splice site selection by components of the spliceosome (i.e. U1 snRNP, U2AF) often based on both the pre-mRNA sequence as well as auxiliary splicing factors. The regulation of splicing is a major pathway in the regulation of gene expression [51], with two distinct but cooperative classes of regulatory elements. The first class is defined by RNA *cis*-acting regulatory sequences, such as 5ʹss and 3ʹss, branch point (BP), intronic and exonic splicing enhancers (ISE, ESE) and silencers (ISS, ESS) [52]. Proteins define the second class, with *trans*-acting splicing factors such as SR (Ser-Arg rich) and hnRNP proteins, which bind to *cis*-acting regulatory sequences in the pre-mRNA [53]. Depending on the position of *cis*-acting regulatory elements with respect to the 5ʹss, *trans*-acting splicing factors can act as activators or repressors [54]. Many *trans*-acting splicing factors are not ubiquitous but are expressed in specific tissues, during development or upon external triggers. Regulation of alternative splicing through tissue-specific *trans*-acting splicing factors plays an essential role in rewiring downstream protein interaction networks, which is crucial for cell differentiation and organ development [55].

Regulation of alternative splicing is widespread, and numerous examples exist in the literature [56]. We illustrate this diversity with a few selected cases. As a first example, temperature-dependent effects in gene expression can be linked to alternative splicing, since temperature-sensitive CDC-like kinases (CLKs) phosphorylate SR-proteins at lower temperatures. This post-translational modification of the SR-protein splicing factors in turn triggers changes in splicing patterns, with wide implications in circadian, tissue-specific and disease-associated settings [57]. Physiological splicing patterns may also be hijacked by viruses, such as in the suppression of interferon response upon viral infection [58]. Recent insights also highlight the significance of splicing and isoform-level regulatory mechanisms in promoting an effective immune response to vaccines [59]. In addition to the interplay between *cis*-acting sequences and *trans*-acting splicing factors, alternative splicing also integrates genetic and epigenetic factors such as transcriptional elongation, DNA methylation, chromatin architecture and histone modifications to finely tune gene expression patterns in a co-transcriptional context [56]. Finally, pre-mRNA modifications including but not limited to N6-methyladenosine (m^6^A), N1-methyladenosine (m^1^A), 5-methylcytosine (m^5^C), pseudouridine (Ψ) and ribose-methylation (2’-O-Me) can contribute to the regulation of gene expression [60]. For instance, a recent report indicates that m^6^A methylation of a 3ʹss can block recognition by the essential splicing factors, hence inhibiting splicing [61].

## Intrinsic features of the 5’-splice site that modulate U1 snRNP binding

**Figure 3.**
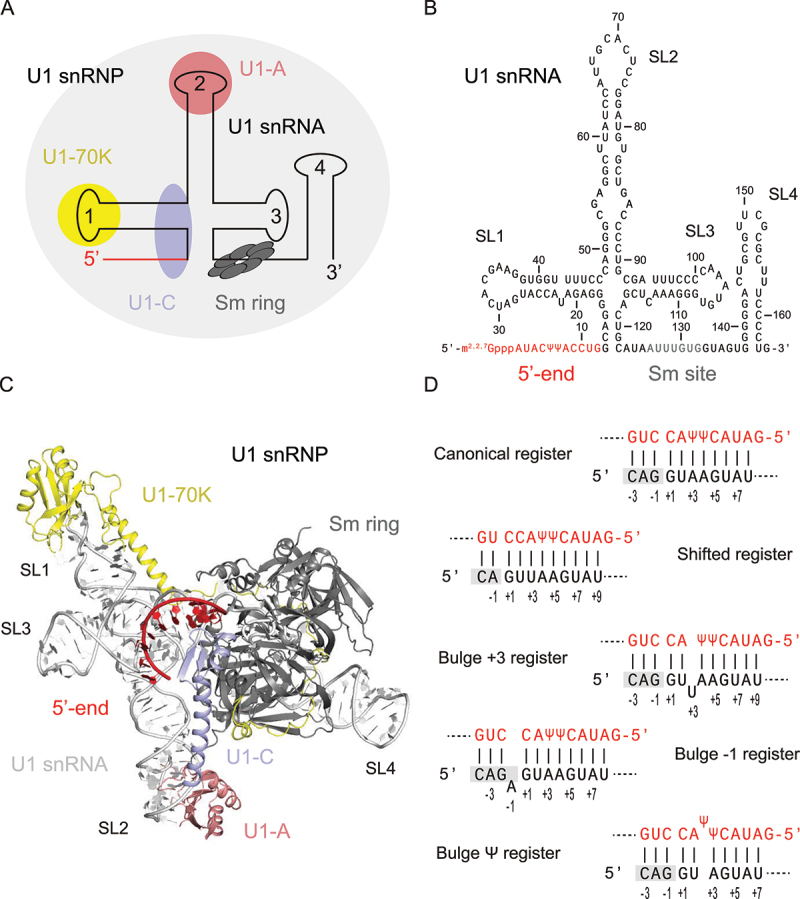
U1 snRNP structure and 5’-splice site (5ʹss) recognition. (a) Summary of U1 snRNP topology. U1 snRNP (light grey) is composed of the U1 snRNA (line), seven Sm proteins (dark grey) and three U1 snRNP specific proteins. (b) U1 snRNA sequence and topology. In U1 snRNA (164 nt), the 5’-end (red) binds to the 5ʹss, the downstream region contains SL1-3, followed by the Sm-site (grey), and SL4 in 3’-end. (c) Structural model of the complete U1 snRNP based on the crystal structure of human spliceosomal U1 snRNP [62]. U1 snRNA (light grey) and its 5’-end (red), the Sm-ring (grey) and U1-specific proteins U1-70 K (yellow), U1-A (pink) and U1-C (purple) are highlighted. (d) Base-pairing registers. U1 snRNA (red) and the 5ʹss (black) can adapt canonical and alternative registers.

U1 snRNP initiates the splicing reaction upon recognition and binding to the 5’-splice site (5ʹss) to define each exon–intron junction within the pre-mRNA. U1 snRNP consists of the U1 snRNA (164 nt), the seven Sm proteins (Sm-B/Sm-B’, Sm-D1, Sm-D2, Sm-D3, Sm-E, Sm-F, and Sm-G), and three U1-specific proteins (U1-A, U1-C, U1-70 K) [62] (Fig. 3a). The U1 snRNA topology consists of an unpaired 5’-end, a four-way junction of three stem-loops (SL1-3) in a trefoil fold, a Sm site, and a fourth stem-loop at the 3’-end (SL4) (Fig. 3b). With respect to U1 snRNA, U1-C, U1-70 K and U1-A are bound to the 5’-end, SL1 and SL2, respectively. The Sm proteins are arranged in a heptameric ring structure around the Sm site [62] (Fig. 3c). In this section, we describe how the unpaired 5’-end of U1 snRNA can recognize a variety of 5ʹss through specific base-pairing registers, prior to discuss to which extent the intrinsic features of the 5ʹss can drive the recognition by U1 snRNP.

### U1 snRNA/5’-splice site base-pairing registers

In mammals, the canonical mechanism of 5ʹss recognition by U1 snRNP relies on base-pairing between the 5’-end of U1 snRNA (5’-m^2,2,7^G_ppp_AUACΨΨACCUG, with Ψ standing for pseudouridine) and the consensus 5ʹss sequence CAG|GUAAGU defined over 9 nucleotides from position −3 to +6 with respect to the exon–intron junction [15] (Fig. 3d). However, the 5ʹss in pre-mRNA only rarely represents an exact matching sequence, but is instead highly degenerate with strict sequence conservation limited to the ubiquitous G_+1_ and U_+2_ nucleotides [63]. Within the 5’ end of U1 snRNA, it is suggested that the presence of pseudouridine (Ψ) bases provides an advantage in 5ʹss discrimination [64]. This RNA modification may be required for the stability of the U1 snRNA interaction with 5ʹss, such as in the case of HIV-1 SD4 RNA with Ψ-G base pairing [65]. The general helical U1 snRNA:5ʹss duplex can accommodate alternative registers that include shifted basepairing and non-canonical base-pairing with bulged nucleotides on either the 5ʹss or U1 snRNA strand (i.e. bulge registers) [63,66,67]. Notably, statistical analysis demonstrated that bulge registers are associated with increased alternative 5ʹss selection, yet with a mechanistic basis that remains to be investigated [63]. Furthermore, base pairing between the U1 snRNA and the 5ʹss is sometimes limited by the accessibility of the 5ʹss itself that can be sequestered into inhibitory secondary structure. This strategy is commonly used to regulate alternative splicing of cassette exons such as SMN2 exon 7 [68] or Map/Tau exon 10 [69]. The sequence of the 5ʹss as well as its accessibility are therefore two major components that influence the 5ʹss strength and usage. In the context of this review, the 5ʹss strength relates to the Gibbs free energy change (ΔG) upon formation of the RNA duplex between the 5’-end of U1 snRNA and the 5ʹss, while the 5ʹss usage relates to a splicing event actually occurring at the splice site.

### Relevance of the 5’-splice site consensus

The diversity of base-pairing registers explains why the conservation of GU-flanking sequences with respect to 5ʹss consensus is not a reliable predictor of splice site strength or usage, thus complicating *de novo* predictions of alternative 5ʹss selection. Instead, empirical evidence suggests that 5ʹss selection is correlated with the binding free energy of U1 snRNA:5ʹss base-pairing for *strong* sites only, in sharp contrast with *intermediate* and *weak* splice sites that cannot be distinguished based on this single descriptor [64]. In terms of a general trend, 5ʹss recognition tends to be positively affected by increased base-pair complementarity with the 5’-end of U1 snRNA [70]. It is unclear how an extended complementarity between the segments flanking the 5’-end of U1 snRNA and the 5ʹss could impact splice site recognition and usage. It is suggested that extensive complementarity promotes 5ʹss recognition but leads to an excessively stable U1 snRNA:5ʹss complex, which would be inhibitory to downstream splicing steps [71]. However, it was also reported that an extended complementarity does not decrease splice site usage, but rather increases 5ʹss recognition and exon inclusion [70]. Despite opposite observations on 5ʹss usage, the positive impact of an extended complementarity on 5ʹss recognition is acknowledged on both sides, showing that increased recognition is not necessarily correlated with increased splice site usage.

The highly degenerate nature of 5ʹss sequences, in combination with the modifying influence of nearby *cis*-acting regulatory motifs, makes it difficult to model relationships between splice site sequence, recognition by U1 snRNA, and splicing efficiency. To understand the relevant numbers involved in these predictions, we can use an example analysis of a 9-nucleotide template of 5ʹss. In such a model system, with nucleotides constrained to NNN|GYNNNN with *N* ∈ {*A,U,G,C*}, the set of all unique 5ʹss would contain 7^4^ or 32768 sequences. In a fascinating work, Wong and co-workers released the quantitative activity profile of all unique 5ʹss measured in human cells by using the method of Massively Parallel Splicing Assay (MPSA) with three distinct gene contexts (*BRCA2* intron 17, *SMN1* intron 7, *IKBKAP* intron 20) [72]. They found that the splice site sequence is a major determinant of 5ʹss recognition and usage within a given gene context. However, differences are substantial across gene contexts with the same sequence, indicating that the splice site sequence alone is indeed not a reliable descriptor of 5ʹss recognition and usage across multiple gene contexts. Nonetheless, subsets of splice-site sequences or patterns were found to correlate with splicing efficiency across different contexts, hence supporting the existence of context-independent models. For instance, only a minor subset of all GC-based 5ʹss sequences were recognized as functional, supporting that U > C substitution at position +2 may lead to suboptimal sequence for base-pairing with U1 snRNA [72]. Based on *BRCA2* and *SMN1* studies, the pattern G_−1_ … G_+5_ was highly preferential, with a single substitution at either position resulting in lower usage, and non-G substitutions at both positions being highly unfavourable. From this exhaustive search, it is speculated that G-C base-pairing at the −1 and +5 positions may contribute to the strong dependency observed [72].

### Computational 5’-splice site prediction

Splice sites define exon/intron boundaries. Therefore, the accurate *de novo* prediction of splice sites is useful for the annotation of genes, and also to find alternative splice sites associated with diseases. In *de novo* prediction tasks, consensus sequences based on Position-Weight Matrix (PWM) are not appropriate because they assume statistical independence between positions, which is inaccurate. In the 1980ʹs, computational approaches based on Artificial Neural Networks (ANNs) first appeared in the RNA field to distinguish translational initiation sites, and outperformed consensus-based methods [73]. Among other computational objects that aim to predict splice sites, many Deep Neural Networks (DNNs) with similar architecture were recently released [74–80]. In these tools, the internal representation of the nucleotide sequence is a chain of characters. Instead, the recent use of evolutionary related sequences and multiple sequence alignment (MSA) was crucial to solve other sequence-related problems in biology [81]. Thus, further development in pre-mRNA sequence representation will undoubtedly propel DNN-based prediction of splice sites to the next level, as seen for other sequence-related problems [81].

## Stabilization of U1 snRNP on weak 5’-splice sites *via* protein–protein interactions

While the 5’-end of U1 snRNA recognizes and base-pairs with the 5’-splice site (5ʹss), the recruitment of U1 snRNP to the 5ʹss is also regulated through direct interactions between U1-specific proteins (U1-70 K, U1-A, U1-C) and splicing factors. This mechanism is particularly common when the 5ʹss sequence is considered to be weak in strength. The auxiliary *trans*-acting splicing factors bind *cis*-regulatory elements near to the splice site using RNA binding domain(s) and contact the U1 snRNP though protein–protein interactions. The additional interactions provide a secondary link to the pre-mRNA that can result in increased recruitment of the 5ʹss recognition machinery onto otherwise weak splice sites. As detailed below, examples of *trans*-acting splicing factors have been found to participate in protein–protein interactions with each of the three essential U1-specific proteins.

### Via U1-70 K

**Figure 4.**
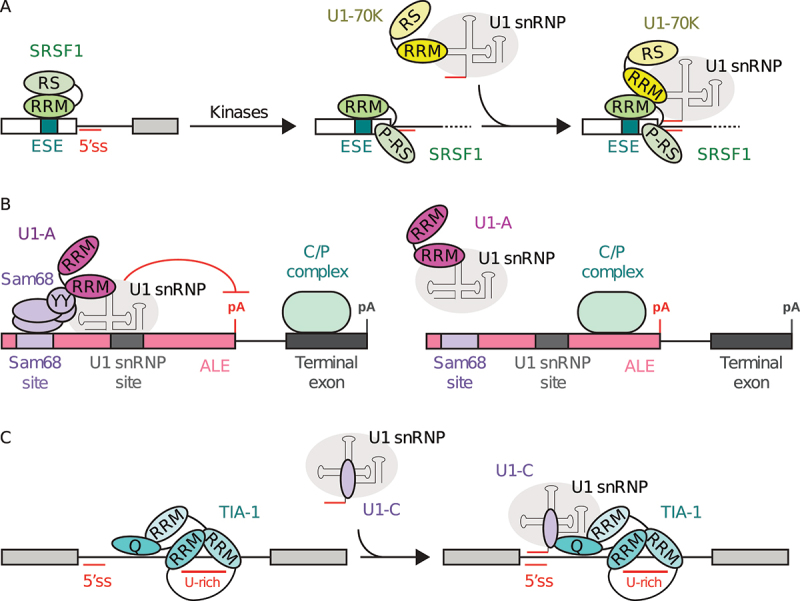
U1 snRNP-specific proteins interact with splicing factors to modulate 5’-splice site (5ʹss) recognition and spliceosome assembly. (a) Recruitment of U1 snRNP at the 5ʹss by SRSF1 upon interaction with U1-70 K [82]. The phosphorylation of ESE-bound SRSF1 in the RS domain makes its RRMs available for interaction with U1-70 K RRM, which contributes to recruiting U1 snRNP to the 5ʹss. (b) Sam68 interaction with U1 snRNP is mediated by U1-A to affect the definition of the alternative last exon [87,89]. The interaction between U1-A RRM1 and Sam68 YY-domain stabilizes the binding of U1 snRNP to the pre-mRNA, which in turn represses the polyadenylation signal and leads to inclusion of the terminal exon. In contrast, the absence of Sam68 does not allow U1 snRNP binding, which results in the inclusion of alternative last exon. (c) Recruitment of U1 snRNP at the 5ʹss by TIA-1 upon interaction with U1-C [94,95]. The binding of TIA-1 RRM1-2 to U-rich sequences downstream of the 5ʹss facilitates the recruitment of U1 snRNP through interactions between TIA-1 Q-rich domain and the U1-C protein.

Serine/arginine-rich splicing factors (SR-proteins) are *trans*-acting splicing enhancers with a shared topology that includes an N-terminal region with one or two RNA binding domains and a C-terminal RS domain. In general, SR-proteins bind to exonic splicing enhancer (ESE) sequences on the pre-mRNA through their RNA binding domains that belong to the RNA recognition motif (RRM) or zinc finger (Znf) families. A secondary interaction is then primarily within U1 snRNP to aid in its recruitment to the 5ʹss. As a specific example, the SR-protein SRSF1, which contains two RRM domains, can recruit U1 snRNP at the 5ʹss through its interaction with U1-70 K [82] (Fig. 4a). The U1 small nuclear ribonucleoprotein 70 kDa (U1-70 K) contains an N-terminal disordered region, followed by an RRM domain that binds U1 snRNA SL1, and a C-terminal RS domain [62]. It was initially thought that the interaction between SRSF1 and U1-70 K was solely mediated by the C-terminal RS domains found in both proteins, and the phosphorylation of SRSF1 RS domain was shown to be crucial for U1 snRNP recruitment to the 5ʹss [83–86]. Recent reports indicate that the RRM domains of SRSF1 bound to the pre-mRNA could also interact with the RRM domain of U1-70 K, hence bridging the whole U1 snRNP to the pre-mRNA, near to the 5ʹss. This interaction is dependent on the phosphorylation state of SRSF1, because its RRM domains can only participate in intermolecular interactions after hyper-phosphorylation of the RS domain [82]. The U1-70 K mediated recruitment of U1 snRNP to the pre-mRNA by SRSF1 is proposed to seed spliceosome assembly.

### Via U1-A

Modulation of U1 snRNP 5ʹss selectivity can also rely on the U1 small nuclear ribonucleoprotein A (U1-A) protein, which contains an N-terminal RRM domain (RRM1) bound to U1 snRNA SL2, followed by a long disordered segment and a C-terminal RRM domain (RRM2). U1 snRNP can be recruited to the 5ʹss by the Src associated in mitosis 68 kDa (Sam68) protein, which contains a STAR domain that binds to U(U/A)AA direct repeats in the pre-mRNA, and a C-terminal YY domain shown to interact with the RRM1 domain of the U1-A protein [87]. Sam68 regulates the alternative splicing of the mammalian target of rapamycin (mTor), and the interaction between Sam68 and U1-A promotes U1 snRNP recruitment to the 5ʹss in intron 5 of mTor pre-mRNA [88]. Disruption of the U1-A/Sam68 interaction through mutation of Sam68 or the *cis*-regulatory element abrogates U1-A mediated recruitment of U1 snRNP at the 5ʹss and splicing [87]. In meiotic cells, Sam68 is highly expressed and regulates alternative last exon (ALE) splicing events in genes required for spermatogenesis [89]. Sam68-regulated ALEs are characterized by the proximity between U1 snRNP and Sam68 binding motifs, and the recruitment of U1 snRNP to Sam68-regulated ALEs is impaired in Sam68^−/−^ germ cells [89]. Upon Sam68 interaction with U1-A, the recruitment of the whole U1 snRNP near to internal polyadenylation sites prevents their recognition by the cleavage and polyadenylation (C/P) complex, abolishing premature transcript termination [89] (Fig. 4b). Overall, Sam68 modulates U1 snRNP recruitement at the 5ʹss through U1-A in a wide range of contexts [87,89].

### Via U1-C

While the topology of U1 snRNP is widely conserved in Eukarya, notable variations of U1 snRNP composition can be observed between phyla. The U1 small nuclear ribonucleoprotein C (U1-C) contains an N-terminal zinc finger (Znf) domain and a long C-terminal tail. U1-C binds to the U1 core domain in an U1-70 K-dependent fashion, while the helix A of U1-C binds in the minor groove of the U1 snRNA/5ʹss RNA duplex [15]. In yeast, Nam8 is a constitutive U1-specific protein, and is composed of three RRM domains (RRM1-3) and a C-terminal Q-rich domain [90,91]. TIA-1 is the human homolog of yeast Nam8, although TIA-1 is not part of human U1 snRNP. In the yeast U1 snRNP, the C-terminal moiety of Nam8 interacts with the C-terminal domain of U1-C, while the N-terminal RRM domains of Nam8 binds to U-rich sequences on the pre-mRNA, which helps recruit yeast U1 snRNP to weak splicing sites [92,93]. In humans, TIA-1 enhances splicing of the K-SAM alternative exon that depends on U-rich intronic splicing enhancer sequences (IAS1) immediately downstream the 5ʹss, and in a U1 snRNP-dependent manner [94]. While not part of human U1 snRNP, TIA-1 still interacts with U1 snRNP through the U1-C protein. The Q-rich domain of TIA-1 makes a direct contact with the N-terminal region of U1-C, enhanced by contacts with the RRM1 domain, while the RRM2/3 domains bind the pre-mRNA [95]. The RRM1 and Q-rich domain of TIA-1 mediates the association with U1 snRNP, and both are required to facilitate its recruitment to the 5ʹss [95]. Consistently, TIA-1 is proposed to bind U-rich sequences downstream the 5ʹss of target exons and to recruit U1 snRNP by contacting U1-C [94,96,97] (Fig. 4c).

## Stabilization of U1 snRNP on weak 5’-splice sites *via* protein–RNA interactions

**Figure 5.**
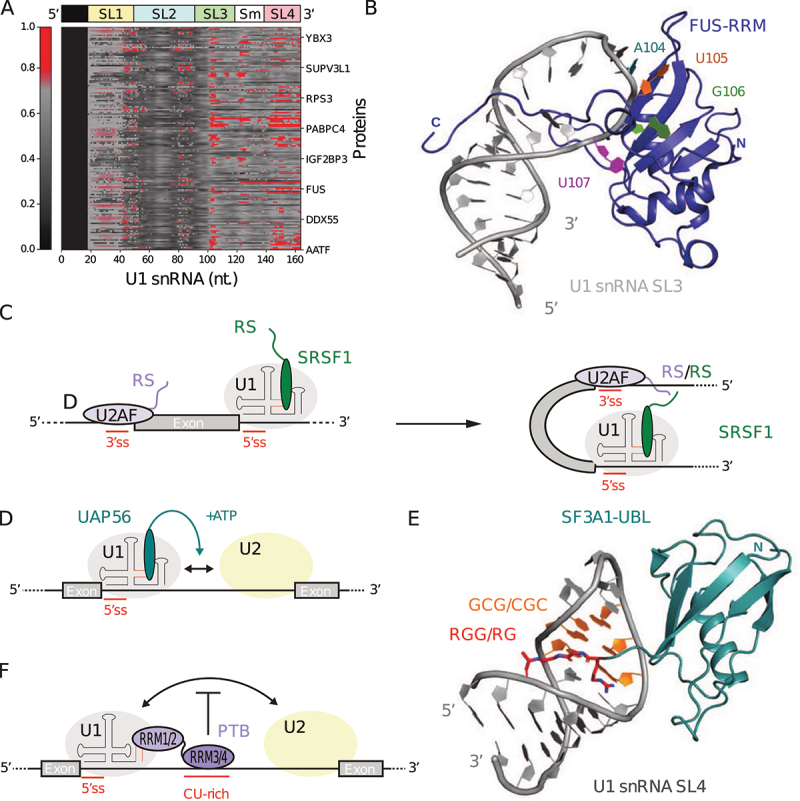
U1 snRNA SL3 and SL4 are targeted by splicing factors to modulate 5’-splice site (5ʹss) recognition and spliceosome assembly. (a) Protein cross-links to U1 snRNA *in vivo* [98,99]. The heat map shows the distribution of cross-links to U1 snRNA for 147 RNA-binding proteins. (b) Solution structure of FUS-RRM (blue) in complex with U1 snRNA SL3 (grey) (pdb code: 6SNJ) [101]. The structure corresponds to the lowest energy model from the NMR ensemble. (c) Model for exon independent recruitment of SRSF1 by U1 snRNP [98]. (d) Model for UAP56 mediated splicing enhancement [100]. (e) Crystal structure of SF3A1-UBL (cyan) in complex with U1 snRNA SL4 (grey) (pdb code: 7P0V) [108]. (f) Model for PTB mediated splicing repression [110,111].

Although the 5’-end of U1 snRNA is the structural segment that actually recognizes and base-pairs with the 5’-splice site (5ʹss), other U1 snRNA segments contribute to splicing regulation as sensors and interaction platforms for signalling. Within U1 snRNP, the stem-loops 3 (SL3) and 4 (SL4) are not bound to proteins and remain exposed to solvent, hence they are available for interactions with splicing modulators (Fig. 3c). The structure of U1 snRNP bound to a short mRNA fragment suggests that SL3 is oriented towards the exon while SL4 faces the downstream intron [17,62]. The analysis of the massive amount of cross-linking and immunoprecipitation (CLIP) data generated by the ENCODE project suggests that U1 snRNA SL3 and SL4 are targets for a number of RNA-binding proteins *in vivo*, and that competitive binding for these two stem loops would be a major determinant of splicing outcomes in mammalian cells [98,99] (Fig. 5a).

### Role of U1 snRNA SL3

The flexible U1 snRNA SL3 includes a 9 base-pair long stem with a single cytosine bulge and a 7 nt loop (Fig. 3b). Mutations in U1 snRNA SL3 are known to disrupt splicing events, highlighting the functional significance of SL3 in the context of U1 snRNP [100]. In the next paragraphs, we provide examples of interactions between the U1 snRNA SL3 and protein partners and discuss how they contribute to 5ʹss selection or spliceosome assembly.

#### FUS binds U1 snRNP to modulate 5ʹss selection via U1 snRNA SL3

The interaction between U1 snRNA SL3 and the RNA-binding protein Fused in Sarcoma (FUS) is particularly relevant for disease. Mutations causing Amyotrophic Lateral Sclerosis (ALS) in FUS are reported to result in aberrant contacts with cytoplasmic U1 snRNA at the Sm site, causing disruption of snRNP biogenesis [101]. FUS is a *trans*-acting splicing factor with a versatile and context-dependent impact on splicing regulation. Physiological and pathological RNA targets of FUS were recently identified using CLIP experiments. FUS strongly associates with pre-mRNAs, and its major nuclear RNA target is the U1 snRNA that is bound by FUS on SL3 [101]. In the solution structure of FUS in complex with a segment of U1 snRNA SL3, the RRM domain interacts with the apical region of U1 snRNA SL3 [101] (Fig. 5b). In this context, the RRM and zinc finger domain of FUS could recognize RNA elements separated by up to 80 Å using a bipartite RNA binding mode [102]. These results suggest that FUS could help position U1 snRNP on weak 5ʹss to modulate RNA splicing and repress premature polyadenylation [101].

#### Exon-independent recruitment of SRSF1 and exon definition

In the early stages of spliceosome assembly, U1 snRNA SL3 interacts with the SR-rich Splicing Factor 1 (SRSF1), a global *trans*-acting splicing enhancer [82]. Classically, SRSF1 is recruited by *cis*-acting exonic splicing enhancer (ESE) sequences, hence contributing to splice site selection by promoting U1 snRNP recruitment. While the interaction of SRSF1 with U1 snRNP has been known for decades, it has previously been attributed to protein–protein interaction between SRSF1 and U1-70 K [82]. Recently, it was demonstrated that SRSF1 binds to U1 snRNP *in vitro* and that SRSF1ΔSR retains interaction capabilities with U1 snRNP [98]. Using U1 snRNA SL3 alone, binding was still observed, and interaction surface mapping on SRSF1 showed that the RRM1 domain is bound to the CA motif at the 5’ side of the loop, while the RRM2 domain binds the 3’ side of the stem [98,103,104]. Consistently, an original and ESE-independent mechanism for SRSF1 recruitment was proposed, in which a single molecule of SRSF1 can be recruited by U1 snRNP at the 5ʹss through contact mediated by U1 snRNA SL3 [98]. From this perspective, SRSF1 binding to U1 snRNA SL3 enables RS domain interaction between SRSF1 and U2AF complex, facilitating the transition from the spliceosomal E complex to the A complex (Fig. 5c). Finally, the analysis of protein cross-links to U1 snRNA *in vivo* revealed that SRSF7 and SRSF9 show similar crosslink distribution as compared to SRSF1, suggesting that the interplay with U1 snRNA SL3 may be shared among a subset of SR-proteins [98].

#### UAP56 facilitates the transition from spliceosomal E to A complex

During spliceosome assembly, the transition from the spliceosomal E complex to the A complex can also be promoted by U1 snRNA SL3 interaction with the 56 kDa U2AF65-Associated Protein (UAP56), a DExD/H-box family RNA helicase involved in mRNA nuclear export and pre-spliceosome assembly. U1 snRNA SL3 interacts with UAP56 in an ATP-dependent manner, facilitating contact between U1 and U2 snRNP and the conversion to the spliceosomal A complex [100] (Fig. 5d). Addition of excess free SL3 in *trans* enhances splicing upon binding to endogenous UAP56 [100]. UAP56 knockdown or U1 snRNA SL3 mutations are phenotypically equivalent, which results in reduction of exon inclusion and lowered splicing efficiency [100]. The helicase activity of UAP56 may also facilitate the melting of SL3 and the binding of SRSF1. However, sequential binding of proteins or competition for binding on SL3 has not yet been explored experimentally.

### Role of U1 snRNA SL4

The rigid stem-loop 4 (SL4) at the 3’-end of U1 snRNA is required for splicing to occur [105]. U1 snRNA SL4 includes a 5 base-pair stem followed by a 2 nt internal loop, a 3 base-pair GCG stem, and an apical UUCG structured tetraloop [106, 107] (Fig. 3b). Herein, we discuss how the interactions between splicing factors and the U1 snRNA SL4 can promote or inhibit the transition from spliceosomal E to A complex.

#### SF3A1 promotes spliceosomal E to A complex transition

U1 snRNA SL4 is a target of the splicing factor 3 subunit 1 (SF3A1) protein. SF3A1 is a constitutive component of U2 snRNP and interacts with U1 snRNA SL4 within the pre-spliceosomal complex A by mediating contact between the 5ʹss and 3ʹss [109]. The N-terminal RRM domains (RRM1/2) of PTBP1 interact with U1 snRNA SL4, while the C-terminal RRM domains (RRM3/4) bind downstream of the 5ʹss [110,111]. The direct interaction between U1 snRNA SL4 and PTBP1 was recently confirmed *in vitro* between intact U1 snRNP and either full-length PTBP1 or just the N-terminal RRM domains (RRM1/2) [111]. In the context of the *c-src* N1 exon, U1 snRNP is known to recognize the 5ʹss, but the presence of PTBP1 results in exon skipping [112]. The binding of PTBP1 to CU-rich motifs downstream of the 5ʹss, and the formation of a ternary complex with nearby U1 snRNP, is thought to prevent contact between U1 and U2 snRNP to inhibit spliceosomal complex A formation [110] (Fig. 5 F). The recent structure of SF3A1-SL4 suggests that PTBP1 and other RNA binding proteins may compete with the ubiquitin-like domain of SF3A1 for the binding to SL4. Thus, U1 snRNA SL4 represents a hotspot for splicing decisions.

### Synergistic role of U1 snRNA SL3/4

Together, U1 snRNA SL3 and SL4 also have synergistic roles in maintaining U1 snRNP function, in regards to cross-intron contact with U2 snRNP to drive exon definition [98,100]. As a consequence, the interaction between U1 snRNA SL4 and the U2 snRNP protein SF3A1 is sensitive to events occurring on SL3, such as mutations or knockdown of the SL3 partner UAP56, which abrogates SL4 interaction with SF3A1 [100]. The analysis of protein cross-links to U1 snRNA *in vivo* also suggests that U1 snRNA SL3 and SL4 are preferential targets for a large number of RNA-binding proteins [98,99] (Fig. 5a). Overall, U1 snRNA SL3 and SL4 are major hubs for splicing regulation through their role in modulating 5ʹss selection, which contributes to exon definition and the transition from the pre-spliceosomal E complex to the A complex in the early stage of the spliceosome assembly.

## Altered 5’-splice site selection and diseases

Alternative splicing needs to be tightly regulated to produce isoforms at the correct time and place, and any deviation can thus cause disease. As discussed in the introduction, alternative splicing is a key modulator of gene expression and can occur through distinct mechanisms including alternative 5ʹss and/or 3ʹss selection [50]. Due to the versatility and intricacy of splicing, patterns of mature mRNA transcripts are highly sensitive to single nucleotide polymorphisms (SNP) when they occur within *cis*-acting regulatory sequences, which explains why defects in RNA splicing have been associated with ~35% of diseases caused by inherited or somatic mutations [113]. It is estimated that ~10% of all disease-causing mutations impact either a 5ʹss or 3ʹss and consistently result in exon skipping, intron retention, or alternative splice site activation [113]. The online database DBASS documents new exon boundaries induced by pathogenic mutations in human disease genes [114]. In the following paragraph, we discuss how inherited diseases and cancers are linked with altered 5ʹss selection by U1 snRNP.

### Spinal muscular atrophy (SMA)

Polymorphism at a *cis*-acting regulatory sequence can modify U1 snRNP 5ʹss selectivity. One of the most studied inherited diseases that falls into this category is spinal muscular atrophy (SMA), the leading genetic cause of infantile death [115]. SMA is an autosomal recessive neuromuscular disease characterized by a progressive degeneration of motor neurons in the spinal cord, leading to muscle weakness and atrophy [115]. More than 95% of SMA cases are caused by a homozygous inactivation of the *SMN1* gene, which encodes for the Survival Motor Neuron (SMN) protein [116]. However, a paralogous *SMN2* gene is also present in the human genome, and a positive correlation is found with a high copy number of the paralogous *SMN2* gene accompanying milder SMA phenotypes [117]. Unfortunately, within the *SMN2* gene a C > T mutation at position 6 of exon 7 weakens the 5ʹss, which results in exon 7 skipping for ~85% of spliced transcripts and the production of a truncated, non-functional SMN protein [118]. Because *SMN2* is the sole source for SMN protein expression in SMA patients, the small fraction of functional SMN that is still produced is not sufficient to ensure physiological function, but it does explain the relationship between *SMN2* copy number and the severity of the disease [117]. Consistently, increasing the amount of functional SMN protein produced by the *SMN2* gene is a therapeutical strategy that was proven to be helpful to reduce the severity of the disease, as discussed later in the review.

### Huntington disease (HD)

Also linked with U1 snRNP activity, Huntington disease (HD) is a rare and inherited condition that causes degeneration of nerve cells in the brain [119]. This autosomal dominant and progressive neurodegenerative disorder is caused by cytosine–adenine–guanine (CAG) repeat expansions in the exon 1 of the huntingtin gene (*HTT*), resulting in the production of a mutant huntingtin (mHTT) protein prone to form aggregates [119]. It was proposed that the *trans*-acting factor SRSF6 binds the CAG expansion in *HTT* exon 1, which could either interfere with U1 snRNP protection of polyA signals or negatively regulate the 5ʹss of intron 1, resulting in a short transcript that includes intron 1 and leads to pathological mHTT [120]. In this context, lowering the levels of mHTT by promoting the inclusion of a poison exon is a therapeutical strategy currently being investigated.

### Familial dysautonomia (FD)

Related to the impairment of 5ʹss recognition, familial dysautonomia (FD) is an inherited autosomal recessive disorder that affects the nerve fibres, causing difficulties to feel pain, temperature, skin pressure, among other related issues [121]. FD is caused by the intervening sequence 20 (IVS20) +6 T > C splicing mutation of the inhibitor of kappa light polypeptide gene enhancer in B-cells, kinase complex-associated protein (*IKBKAP*) gene, causing abnormal exon 20 skipping in the mature mRNA due to weakened recognition of the mutant 5ʹss by U1 snRNP [122]. The loss of exon 20 leads to a frame shift and reduced expression of IKAP protein, compromising tRNA modification and neuronal cell survival [121]. Herein, restoring exon 20 inclusion with splicing modifiers that promotes 5ʹss recognition is a therapeutical strategy under investigations [123].

### Hutchinson–Gilford Progeria Syndrome (HGPS)

Point mutations can sometimes create a stronger 5ʹss, like in the rare Hutchinson–Gilford Progeria Syndrome (HGPS). Occurring mainly in patients without familial history, HGPS is an autosomal dominant condition resulting from mutations in the lamin A gene (*LMNA*), which causes premature ageing and early death from related complications [124]. In ~90% of cases, HGPS is caused by a *de novo* point mutation C > T in position 1824 of the *LMNA* gene, within the exon 11, which creates an alternative 5ʹss and results in the production of progerin, a partially deleted form of nuclear lamin A responsible for dysfunctional nuclear membrane and premature senescence [125]. In preclinical studies, it was shown that the pharmacological blockade of the *LMNA* splice site leading to progerin production was a promising treatment approach for patients affected with HGPS [126].

### Emerging role in cancer and global relevance

Finally, deregulation in alternative splicing is increasingly reported to play an important role in tumorigenesis [127]. Aberrant splicing can lead to loss of function in tumour suppressors or activation of oncogenes, which explains how alternative splicing deregulation may promote hallmarks of cancer such as increased cell proliferation, metabolism, genomic instability, and more [128]. For instance, deregulation of alternative 5ʹss selection in intron 2 of the *Bcl2L1* gene can increase the ratio between large (Bcl-xL) and small (Bcl-xS) mRNAs that encode for the anti- and pro-apoptotic isoforms, hence contributing to apoptosis inhibition [128]. Many tumour-associated splicing changes arise due to alterations in particular components of the splicing machinery [129,130]. Regarding *cis*-regulatory sequences, the systematic analysis of multiple cancer types revealed that somatic mutations do not only disrupt canonical splice sites, but can also lead to *de novo* splice sites in cancer-related genes such as *TP53, ATRX, BAP1 CTNNB1, RB1*, and more [131]. In addition, recurrent mutation at 5’-end of U1 snRNA were recently correlated with specific tumour types such as Sonic hedgehog (SHH) medulloblastomas, where snRNA-mutant tumours have significantly disrupted RNA splicing patterns [132]. Overall, the relationship between splicing patterns and cancer explains why tumour-associated splicing variants such as Bcl-xL (e.g. lymphoma), Δ4-EGFR (e.g. glioma), Cyclin D1b (e.g. breast cancer), and more, are increasingly proposed as biomarkers in oncology [133].

In summary, mutations in the 5ʹss and other *cis*-acting regulatory sequences have a critical impact in the early stages of spliceosome assembly and associated clinical outcomes. Accordingly, the scientific community has developed synthetic tools to rationally manipulate 5ʹss selection for therapeutic purposes. In the following part, we discuss recent developments of these technologies that have already delivered innovative therapeutic solutions.

## Correction of 5’-splice site selection with antisense oligonucleotides

**Figure 6.**
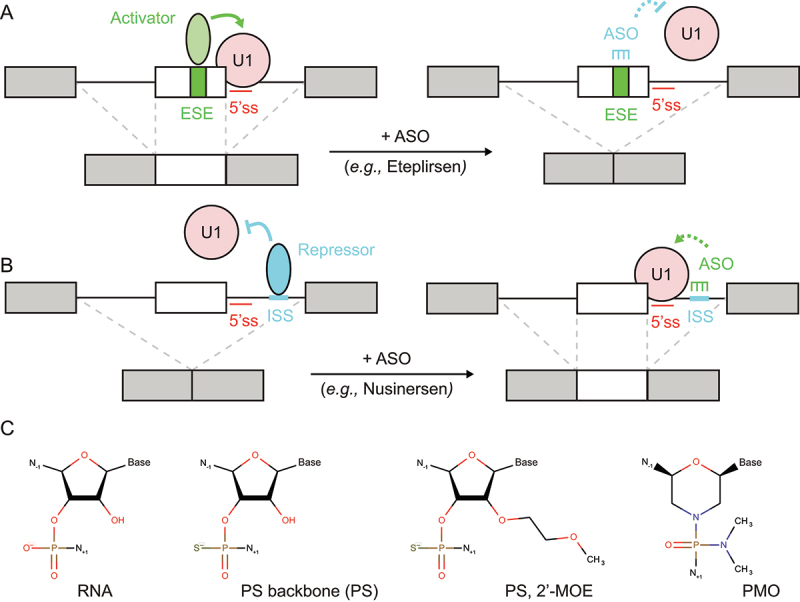
Antisense oligonucleotide (ASO) can modulate 5’-splice site (5ʹss) strength by masking nearby cis-regulatory sequences. (a) Exon skipping. ASO can mask enhancer sequences, promoting exon skipping. (b) Exon inclusion. ASO can mask silencer sequences, promoting exon inclusion. (c) RNA modification. Phosphorothioate (PS) backbone, 2’-O-methoxyethyl (2’-MOE), and phosphorodiamidate morpholino oligomer (PMO).

Antisense therapy is a form of treatment relying on antisense oligonucleotides (ASOs) to target highly specific regions either in the pre-mRNA or mature mRNA. In the context of splicing, ASOs can be directed to mask *cis*-acting regulatory elements in the pre-mRNA, in order to induce appropriate changes in splicing pattern depending on the disease and specific gene context (Fig. 6 (a,b)). ASOs are typically designed to mask enhancer or silencer sequences near a specific 5ʹss, instead of targeting the splice site itself. By masking the regulatory sequence, the ASO prevents splicing factors from being associated with the pre-mRNA, thereby modulating the recruitment of the U1 snRNP to correct the pathological splicing pattern. The next paragraphs provide examples of ASO-mediated exon inclusion for the treatment of spinal muscular atrophy (SMA) or exon exclusion in the case of Duchene Muscular Dystrophy (DMD). We also describe how ASO could eventually be used for patient-customized therapy.

### ASO-mediated exon inclusion

As described previously, in SMA patients the *SMN2* gene produces only residual amount of functional Survival Motor Neuron (SMN) protein that is insufficient to compensate for the loss of *SMN1* [116,117]. Compared to *SMN1*, the *SMN2* gene has a C > T substitution at position 6 of exon 7 which weakens the 5’-splice site (5ʹss), resulting mostly in exon 7 exclusion and therefore a non-functional protein [118]. Based on this observation, splice-switching ASOs (SSOs) were developed in order to shift splicing towards exon 7 inclusion to recover physiological amount of functional SMN protein [134]. The intronic splicing silencer N1 (ISS-N1) was chosen as the target, since it is located immediately downstream of the weak 5ʹss of *SMN2* exon 7 and is the binding site for the *trans*-acting splicing repressor hnRNPA1 [68,135] (Fig. 6a). The abrogation of hnRNPA1 binding to ISS-N1 by SSOs alleviates repression and mechanically promotes 5ʹss recognition and exon 7 inclusion *in vivo* [136–141]. On this premise, the SSO drug Nusinersen (SPINRAZA) was developed and thus became the first treatment approved for SMA [142]. Nusinersen is a 18 nt 2’-*O*-(2-methoxyethyl)-oligoribonucleotide (2’-*O*-MOE) SSO with a fully modified phosphorothioate (PS) backbone (Fig. 6c). The use of 2’-*O*-MOE bases and PS backbone confers resistance to nuclease degradation in the body [143]. Nusinersen is administered into the cerebrospinal fluid (CSF) *via* intrathecal injection with a prolonged half-life of 135–177 days in the CSF. Nusinersen improved neuromuscular functions in infants with SMA and is now used to treat infantile-onset SMA [142].

### ASO-mediated exon skipping

In addition to exon inclusion, SSOs can be directed to promote exon skipping. On the one hand, Duchene Muscular Dystrophy (DMD) is caused by mutations in the *DMD* gene encoding for the dystrophin protein, inducing progressive muscle degeneration and weakness. DMD mutations often result in exon deletion, leading to the creation of premature stop codon and the rapid degradation of dystrophin mRNA by nonsense-mediated decay (NMD) [144]. On the other hand, Becker Muscular Dystrophy (BMD) is a less severe form of dystrophin-related disease. Mutations in the *DMD* gene are still present but do not disrupt the mRNA open reading frame, which results in a non-physiological dystrophin isoform, but partially retains its function [145]. Therefore, SSOs were designed in order to induce exon skipping in DMD genotypes, to prevent the creation of a premature stop codon and to mimic BMD genotypes. The SSO drug Eteplirsen (EXONDYS 51) was developed on this basis and is approved in the USA [146]. Eteplirsen targets dystrophin pre-mRNA and induces exon 51 skipping, which restores the mRNA open-reading frame and results in a shortened, but functional dystrophin. Eteplirsen binds to *cis*-acting exonic splicing enhancer (ESE) sequences in exon 51, which presumably prevents the binding of *trans*-acting splicing activators and subsequent recruitment of U1 snRNP [147] (Fig. 6b). Eteplirsen is a 30 nt SSO with phosphorodiamidate morpholino- (PMO-) based chemical modifications and is indicated for ~14% of all DMD patients. The PMO modification confers resistance to a variety of enzymes, and the drug is administered intravenously on a weekly basis, with a half-life of 3–4 h in the serum [146] (Fig. 6c).

### Patient-customized therapy

Notably, SSOs represent a great opportunity for personalized medicine, with a recent report of patient-customized SSO therapy against ceroid lipofuscinosis 7 (CLN7) [148]. CLN7 is a form of Batten’s disease, a rare and fatal neurodegenerative disease, and the case was a six-year-old girl with symptoms that included blindness, ataxia, seizures, and developmental regression. Genetic profiling revealed a relevant mutation in *MFSD8* gene with G > C substitution at position 1102, while RNA sequencing revealed mis-splicing of exon 6 into a cryptic splice-acceptor site in *MFSD8* intron 6, overall predicting premature translational termination of MFSD8 protein. Based on experience gained from the development of Nusinersen for SMA, SSOs were designed to target enhancer sequences near the cryptic splice-acceptor site. This approach led to the creation of Milasen, a 22 nt SSO with 2’-*O*-(2-methoxyethyl)-oligoribonucleotide (2’-*O*-MOE bases) and a fully modified phosphorothioate (PS) backbone [148] (Fig. 6c). After completion of toxicity studies in animals, Milasen was administered to the patient by intrathecal injection over a year on a monthly basis. Milasen treatment tripled the ratio of normal:mutant MFSD8 protein in the patient’s fibroblasts, which alleviated lysosomal dysfunction. This patient-customized SSO therapy objectively reduced the frequency and duration of seizures in the patient, while demonstrating that ASOs can be used for the future development of personalized medicines.

## Correction of splicing with small-molecule splicing modifiers

While ASO are powerful tools to correct splicing in disease, this class of drugs cannot cross the blood–brain barrier and therefore may require frequent intrathecal injections, such as for treatment of spinal muscular atrophy (SMA). Orally administered, small-molecule splicing modifiers are being actively developed to make 5’-splice site (5ʹss) correcting therapies more accessible to the patient. As detailed below, there are now examples of small drugs created to promote exon inclusion in the treatment of SMA and Huntington disease (HD). General details on how drugs can achieve splicing correction at the molecular level is also discussed.

### Exon inclusion in spinal muscular atrophy (SMA)

**Figure 7.**
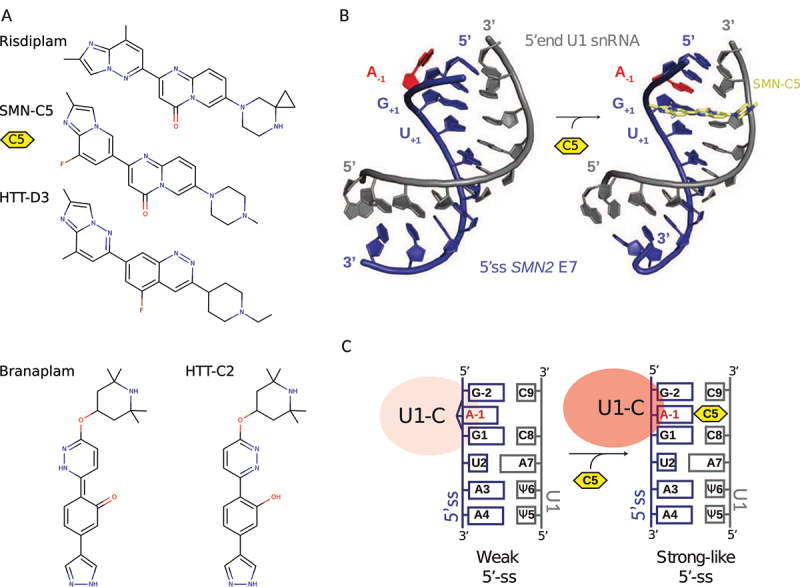
Small-molecule splicing modifiers can strengthen 5’-splice site (5ʹss) interaction with U1 snRNP through the mechanism of bulge-repair. (a) Chemical structure of Risdiplam, Branaplam, and analogues for the treatment of spinal muscular atrophy (SMA) and Huntington disease. (b) Solution structures of U1 snRNA 5’-end (grey) in complex with the 5ʹss (blue) of SMN exon 7, in absence (left, pdb code: 6HMI) and in presence (right, pdb code: 6HMO) of SMNC5 (yellow) [101]. Within the 5ʹss, the A-1 (red) bulging out in absence of small molecule (left) turns inward when SMN-C5 is present (right). (c) The bulge-repair concept. A weak 5ʹss may be strengthened by small-molecule splicing modifiers acting as a glue between U1 snRNP and the 5ʹss.

For SMA, several orally administered small molecules that modify the splicing of *SMN2* gene to promote exon 7 inclusion have been designed [149–151]. Despite their success in restoring effective SMN levels *in vitro* and *in vivo*, the initial molecular scaffolds raised safety concerns as assayed by *in vitro* phototoxicity and mutagenicity (due to coumarins, iso-coumarins) and *in vivo* toxicity upon long-term exposure at high concentration (from pyrido-pyrimidinones) [151]. In response, a pyridazine scaffold was developed to reduce toxicity. Using this scaffold, *branaplam* was developed to stabilize the interaction between the spliceosome and *SMN2* pre-mRNA to promote exon 7 inclusion [152]. *Branaplam* showed efficacy in a mouse model of severe SMA with an increase of full-length RNA and protein levels for SMN, and extended survival [153]. *Branaplam* was the first oral small-molecule splicing modulator tested in SMA Type I patients, but was nevertheless discontinued in phase II/III trials due to approval of similar SMA therapies [154] (Fig. 7a). Among those therapies, drug design efforts led to the discovery of a novel series of SMN-C having a benzamide as a core, which shows an excellent *in vitro* and *in vivo* safety and efficacy profiles on two models of SMA mice (adult C/C-allele and neonatal Δ7) [155]. This approach has led to the newest SMA drug, *risdiplam*, a selective *SMN2* splicing modifier [156] (Fig. 7a). The compound has gone through clinical trials for the treatment of SMA in patients of all ages and stages, and is now approved for therapy [157].

### Pseudoexon inclusion in Huntington disease (HD)

In Huntington disease (HD), one of the key challenges is to ensure optimal delivery and distribution throughout the Central Nervous System (CNS). Therefore, compounds that cross the blood–brain barrier and can be administered orally are a priority for therapy development. As described previously, HD is caused by cytosine–adenine–guanine (CAG) repeat expansions in the huntingtin (HTT) gene, which produces a pathogenic mutant HTT (mHTT) protein [119,158]. In order to lower levels of mHTT, a strategy consists of promoting nonsense-mediated mRNA decay (NMD), a surveillance pathway that eliminates mRNA transcripts that contain premature termination codons. In this context, small-molecule splicing modifiers were designed to promote selective inclusion of a pseudoexon containing a premature termination codon [159]. The selected pseudoexon is located within intron 49 and contains a weak 5ʹss with non-canonical GA dinucleotide at positions −2 and −1; a pattern known to cause inefficient splicing, such as seen in the case of *SMN2* exon 7. The initial lead compound HTT-C2 was selected because it strengthens the non-canonical 5ʹss of the selected pseudoexon, hence introducing a premature stop codon that prevents full-length protein production and promotes mRNA degradation *via* NMD [159] (Fig. 7a). Subsequently, the lead molecule HTT-D3 was developed with improved distribution in the body and was found to result in correlative and equal reduction of mHTT protein levels in plasma and cerebral spinal fluid of Hu97/18 mice [159] (Fig. 7a). Mechanistically, HTT-D3 has a strong preference for the non-canonical AGA|GUAAG 5ʹss, which is similar to the motif recognized by *risdiplam*. Consistently, *branaplam* was shown to be effective in mouse models of HD [160]. Overall, this shows that HTT-C2 and analogues strengthen the interaction between U1 snRNP and the 5ʹss, thus enabling exon definition by the spliceosome [159,161].

### Molecular basis for splicing correction

How splicing modifiers may stabilize the interaction between the pre-mRNA and the spliceosome was further investigated in the context of spinal muscular atrophy (SMA). Recent efforts have determined the mode of action of the highly selective *SMN2* splicing modifier SMN-C5, a chemical analogue of *risdiplam* with comparable efficacy *in vitro* and *in vivo* [161,162]. Combining RNA splicing assays, chemical proteomics, and nuclear magnetic resonance (NMR) spectroscopy, it was found that the drug functions at the molecular level by interacting with a tertiary RNA structure that includes the exonic splicing enhancer (ESE) sequence of *SMN2* pre-mRNA exon 7, with the RNA helix formed by the 5ʹss and the 5’-end of U1 snRNA [162]. The solution structures of the intermolecular RNA helix (5ʹss/U1 snRNA) without and with SMN-C5 were solved by NMR spectroscopy, showing how the splicing modifier stabilizes an unpaired adenine in position −1 at the exon–intron junction in the RNA helix base stack through the *bulge-repair* mechanism [161] (Fig. 7b). An allosteric change caused by SMN-C5 promotes the binding of U1-C zinc finger, and thus whole U1 snRNP, to the 5ʹss of *SMN2* pre-mRNA exon 7 (Fig. 7c). This conformational change thereby converts the otherwise weak 5ʹss to a stronger 5ʹss (Fig. 7c). Altogether, the *risdiplam* analogue SMN-C5 acts as a glue that strengthens the interaction between U1 snRNP and the 5ʹss of *SM2* pre-mRNA, thus promoting exon 7 inclusion [161,162].

## Conclusion

In recent years, ASOs and small-molecule splicing modifiers that target 5ʹss selection by U1 snRNP were approved for therapy [142,146,148,154,157]. Further research in splicing regulation at the 5ʹss will be key to develop innovative drugs against genetic diseases and cancer. Along these lines, there is a strong need for more systematic research to probe the interplay between 5ʹss and other *cis*-acting regulatory elements such as enhancer and silencer sequences within introns and exons. In addition, there is a need to investigate the interactome of U1 snRNP, which includes RNA-binding proteins that are associated with U1-specific proteins and U1 snRNA (*e.g.,* SL3-4), all playing a role in 5ʹss selection and spliceosome assembly. Historically, splicing events were believed to be under the sole dependence of *cis*-acting regulatory motifs on the pre-mRNA, to which *trans*-acting splicing factors would bind and tune splice site recognition and spliceosome assembly. Therefore, the discovery of U1 snRNP as a binding platform that tunes 5ʹss definition is a conceptual breakthrough, since it shows that splicing can be modulated through U1-mediated protein–protein or protein–RNA interactions, sometimes independently from *cis*-acting regulatory sequences [82,87,94,98,100,101,105].

Given the variability of splicing patterns across tissues, between individuals, and along time, the pursuit of a set of formal rules that could predict splicing events from RNA features (i.e. a splicing code) remains largely unsolved. An explicit definition of such a code is made even more difficult by the new role of U1 snRNP as a binding platform for splicing factors. Indeed, the U1 snRNP interactome is now considered to be as essential as RNA features and splicing factors, which complicates the determination of the splicing rules. Nevertheless, the accumulation of experimental data sampling the interplay between gene context and splicing outcomes may eventually provide the basis for an implicit definition of the splicing code. Such analysis could use regressions based on Artificial Intelligence (AI) with supervised learning approaches such as Artificial Neural Networks (ANNs) [74–80]. While the latter do not generally provide a meaningful representation of the relationship between parameters and outcome, they may constitute an implicit definition of the splicing code to more reliably predict splicing events based on RNA features, tissue-specific splicing factors, and proteins that interfere with U1 snRNP. This type of modelling would be beneficial to the development of new ASO drugs and small-molecule splicing modifiers. Indeed, ASO development would take great advantage of reliable prediction tools, based on large ensemble of experimental data, in order to know in advance which RNA region to target in the pre-mRNA that will produce the desired splicing outcome. Similarly, small-molecule splicing modifiers that act as a glue between the 5’-end of U1 snRNA and the 5ʹss, in a fashion termed as *bulge-repair*, may benefit from modelling efforts to identify which particular 5ʹss may be targeted given the molecule [161].

Overall, understanding and correcting pre-mRNA splicing regulation at the 5ʹss provides a fundamental basis for therapy development. ASOs and small-molecule splicing modifiers are effective to treat a growing number of inherited diseases, and they are increasingly considered in oncology where many mutations relevant to splicing were correlated to various types of cancers [127,131]. In this context, the importance of studying U1 snRNP is even raised, because it is the central player to define the 5ʹss.
